# IMRT combined with concurrent chemotherapy plus adjuvant chemotherapy *versus* IMRT combined with concurrent chemotherapy alone in patients with nasopharyngeal carcinoma

**DOI:** 10.18632/oncotarget.14799

**Published:** 2017-01-23

**Authors:** Qiulu Zhong, Xiaodong Zhu, Ling Li, Song Qu, Zhongguo Liang, Fanyan Zeng, Xinbin Pan

**Affiliations:** ^1^ Department of Radiation Oncology, The Affiliated Cancer Hospital of Guangxi Medical University, Cancer Institute of Guangxi Zhuang Autonomous Region, Nanning, Guangxi, China

**Keywords:** nasopharyngeal carcinoma, intensity-modulated radiotherapy, concurrent chemoradiotherapy, concurrent chemoradiotherapy plus adjuvant chemotherapy

## Abstract

**Purpose:**

To evaluate the efficacy of IMRT combined with concurrent chemotherapy followed by adjuvant chemotherapy compared with IMRT combined with concurrent chemotherapy alone in patients with nasopharyngeal carcinoma.

**Methods:**

From January 2007 to December 2014, we collected 797 staged II-IVb [UICC = Union for International Cancer Control criteria (7th edition)] NPC patients for analysis. After 1:1 matching,we selected 261 cases as the CCRT group, another 261 patients as the CCRT+AC group. Using Kaplan-Meier to calculate the overall survival (OS), locoregional failure-free survival(LFFS), distant metastasis failure-free survival(DMFS). The log-rank test and Cox-proportional hazards model to evaluate the prognostic factors.

**Results:**

After matching, there were 261 patients in each group. In CCRT+AC group, The 1-,2- and 3- year os rates were a little higher than in CCRT group(99.6% *vs* 97.9%,97.4% *vs* 96.2%,93.8% *vs* 86.9%, *P* = 0.150). There were no significant difference in 1-,2-,3- year OS, LFFS, DMFS between the two groups. In subgroup analysis, a little higher OS rate in CCRT+AC group for staged III, IV and T4(III:100% vs 100%, 97.6% *vs* 95.8%, 94.0% *vs* 84.0%; IV: 99.1% *vs* 95.4%, 96.3% *vs* 95.4%, 90.5% *vs* 79.4%, *P* = 0.047;T4:99.1% *vs* 95.2%, 97.1% *vs* 95.2%, 90.9% *vs* 78.2%, *P* = 0.055). No significant difference were observed in OS, LFFS,DMFS between the groups.

**Conclusion:**

IMRT combined with concurrent chemotherapy followed by adjuvant chemotherapy might improved 1-,2-,3- year of OS. Whether or not add adjuvant chemotherapy it had similar LFFS rate and DMFS rate in patients with nasopharyngeal carcinoma. Locally advanced NPC patients (III, IV and T4)might benefit from the adjuvant chemotherapy.

## INTRODUCTION

Nasopharyngeal carcinoma (NPC)is popular in China, especially in Guangdong, Guangxi, Hunan, Hainan et al. Radiation therapy is the major treatment for NPC. At present, the standard treatment for NPC is concurrent chemo-radiotherapy. Since 0099 trail [1] reported that conventional chemotherapy combined with concurrent chemotherapy plus adjuvant chemotherapy can improved the overall survival in NPC patients in 1998. This study made sure the efficacy in treated with NPC. Joseph Wee [2] also verified the results as similar as 0099 trail. At the same year, they found that chemotherapy can improve the control rate of distant metastasis . Based on those research, the treatment of conventional radiation therapy combined with concurrent chemotherapy followed by adjuvant chemotherapy became a standard method to treat locoregionally advanced nasopharyngeal carcinoma at North America. But recently, a number of Meta analysis [3, 4] showed that, concurrent chemo-radiotherapy plus adjuvant chemotherapy could not improved the survival rate and most of researches were based on conventional radiotherapy. Now coming into the IMRT era, the problem whether or not adjuvant chemotherapy should be given after finished the concurrent chemo-radiotherapy stills controversial. So we collected 522 staged II-IVb NPC patients who received IMRT combined with concurrent chemotherapy followed by adjuvant chemotherapy or only received IMRT combined with concurrent chemotherapy, to investigate the value of adjuvant chemotherapy in NPC treatment.

## MATERIALS AND METHODS

### Patients

We retrospectively collected a total of 797 stagedII-IVb[UICC = (Union for International Cancer Control criteria (7th edition)] NPC patients at the department of radiation oncology of tumor hospital affiliated with Guangxi Medical University, Nanning PR China, from January 2007 to December 2014. Included 261 cases treated with CCRT and 536 patients treated with CCRT+AC. The inclusion criteria:(1) All the patients were initial treatment who never received any chemotherapy or radiotherapy ;(2)patients aged ranged from 16 to 75 with no distant metastasis ;(3)KPS ≥70 ;(4) pathologic type was non-keratinizing and undifferentiated carcinoma. Those who had radiotherapy, chemotherapy or surgery to the head and neck,or those patients who had double cancer, serious heart and lung diseases,distant metastasis before treatment were exclusion. Before treated, all the patients had a medical history, physical examination, assessment of performance status, complete blood cell count, full bio-chemical profile, EB-virus DNA copies, electrocardiogram, chest computerized tomography (CT), abdominal ultrasound, magnetic resonance imaging (MRI) for nasopharyngeal and neck, nasopharyngeal fiber optic endoscopy and bone scan. In CCRT+AC group,all the participants were received cisplatin plus 5-fluorouracil (PF) as the pattern of adjuvant chemotherapy .Because the KPS of baseline between the two groups were statistically significant difference . So we selected totally 261 patients who received CCRT, we matched 1:1 with the CCRT group on age, sex, overall stage, T classification, N classification, KPS, as the CCRT+AC group. After matched,the baseline data of the two groups balance. (Table 1)

**Table 1:** Baseline patient characteristics in the pre-matching and post-matching cohort

	Pre- matching			post- matching		
group	CCRT^b^	CCRT+AC^c^	P value	CCRT	CCRT+AC	P value
patients	261	536		261	261	
age(year)	47(22-73)	44(16-75)	0.060	47(22-73)	44(16-75)	0.191
sex			0.175			0.250
male	185(70.9)	404(75.4)		185(70.9)	189(72.4)	
female	76(29.1)	132(24.6)		76(29.1)	72(27.6)	
overall stage ^a^			0.251			1.000
II	37(14.2)	73(13.6)		37(14.2)	37(14.2)	
III	103(39.5)	244(45.5)		103(39.5)	103(39.5)	
IV	121(46.4)	219(40.9)		121(46.4)	121(46.4)	
T classification^a^			0.274			1.000
T1	14( 5.4)	36( 6.7)		14( 5.4)	14( 5.4)	
T2	57(21.8)	115(21.5)		57(21.8)	57(21.8)	
T3	76(29.1)	185(34.5)		76(29.1)	76(29.1)	
T4	114(43.7)	200(37.3)		114(43.7)	114(43.7)	
N classification^a^			0.939			1.000
N0	12( 4.6)	25( 4.7)		12( 4.6)	12( 4.6)	
N1	95(36.4)	198(36.9)		95(36.4)	95(36.4)	
N2	141(54.0)	281(52.4)		141(54.0)	141(54.0)	
N3	13( 5.0)	32( 6.0)		13( 5.0)	13( 5.0)	
KPS^d^			0.004			1.000
90-100	191(73.2)	337(62.9)		191(73.2)	191(73.2)	
70-80	70(26.8)	199(37.1)		70(26.8)	70(26.8)	

a By Union for International Cancer Control criteria (7th edition).

b CCRT=concurrent chemoradiotherapy

c AC=adjuvant chemotherapy

d KPS= Karnofsky performance status

The method of matching ,first is selected a total of 261 CCRT patients, then matching 1:1 on age, sex, KPS, overall stage, T classification, N classification with the CCRT group, selected the satisfactory patients from 536 patients in CCRT+AC.

### Radiotherapy

All enrolled patients were received intensity-modulated radiotherapy(IMRT) technique what was administered by a 6 MV-X ray liner accelerator. Patients were immobilized with a head- -neck-shoulder thermoplastic mask in the supine position. Then a CT simulation was carried out to scan the head and neck from the bottom-up. The scan range from the vertex to 2 cm below the clavicle. The slice extending from 2cm over the anterior clinoid process to the level of cartilagines thyreoidea. The slice thickness was 2.5mm, apart from 2.5mm,the remaining were 5mm. We use Precise Plan 2.11(Elekta,Crawley,UK)he Philips pinnacle v8.0(Philips Medical Systems,Milpitas,CA) to delineat the target volumes. The target volumes were delineated according to the International Commission on Radiation Units and Measurements Reports (ICRU)50 and 62.Gross tumor volume in the nasopharynx (GTVnx) stands for what we saw the gross tumor from the clinical examination and imaging examination . Gross

tumour volume of involved lymph nodes(GTVnd) was the positive lymph nodes. Clinical tumor volumes one(CTV1) was the high risk clinical tumor volumes, including GTV plus 5-10mm margin and metastasis lymph nodes. Clinical tumor volumes two (CTV2)was the low risk clinical tumor volumes, including the lymphatic regions and CTV1 plus 5-10mm margins. Planning target volumes(PTV) stand for a 3mm margins were added to the GTV and the CTV. The total radiation prescribed dose was 68-74Gy/30-33 fractions to the PGTVnx,60-71Gy/30-33 fractions to the PGTVnd,60-66Gy/28-30 fractions to the PCTV1, 50-60Gy/28-30 fractions to the PCTV2 at 5 fractions per week during a period of 6-7 weeks.

### Chemotherapy

Concurrent chemotherapy :1 to 3 cycles of Cisplatin alone 100mg/m2 every 3 weeks;

Adjuvant chemotherapy:1 to 3 cycles of Cisplatin 80mg/m2 on day 1 to day 3 and 5-fluorouracil(5-Fu) 750 mg/m2/d on day 1 to day 5 (or continuous intravenous infusion for 120 hours) every 3 weeks (Table 2);

**Table 2:** chemotherapy administrated to 522 NPC patients in the pro-merging groups a DDP= Cisplatin alone

Regimen	Weeks	Group		Pvalue
		CCRT^c^ (n=261)	CCRT+AC^d^ (n=261)	
Concurrent chemotherapy				0.001
DDP^a^	1	17	11	
	2	77	119	
	3	167	131	
Adjuvant chemotherapy				
PF^b^	1	-	70	
	2	-	152	
	3	-	39	

a DDP= Cisplatin alone

b PF= Cisplatin plus 5-fluorouracil

c CCRT=concurrent chemoradiotherapy

d AC=adjuvant cbhemotherapy

But the p value of chemotherapy regimen between two groups had significantly difference,so we merged patients who receiving 2 cycles with 3 cycles in order to reduce the errors.(Table 3)

**Table 3:** chemotherapy administrated to 522 NPC patients in the post-merging groups

Regimen	Weeks	Group		P value
		CCRT^c^	CCRT+AC^d^	
		(n=261)	(n=261)	
Concurrent chemotherapy				0.244
DDP^a^	1	17	11	
	2-3	244	250	
Adjuvant chemotherapy				
PF^b^	1	-	70	
	2	-	152	
	3	-	39	

a DDP= Cisplatin alone

b PF= Cisplatin plus 5-fluorouracil

c CCRT=concurrent chemoradiotherapy

d AC=adjuvant cbhemotherapy

### Follow-up

After all treatment completion, all patients were subsequently followed up every 3 months for the first two years . Every 6 months through the following 3 to 5 years, and then per annum. The follow-up content including body check, complete blood cell count, liver and kidney founctions, EB virus DNA copies, chest computerized tomography (CT), abdominal ultrasound, magnetic resonance imaging (MRI) for nasopharyngeal and neck, nasopharyngeal fiber optic endoscopy and bone scan. The primary endpoint was OS,LFFS,DMFS. The whole group median follow-up period was 25.65 months(range, 2.13-98.70 months).

### Statistical analysis

All data were analyzed by the statistical package for Social Sciences, version 19.0 (SPSS,Chicago, IL.USA). We measured the survival time from the first day the patient diagnosed NPC to the day of the event. Theχ^2^ test was used to compare the based-line data(e.g: sex, KPS, clinical stage, T classification, N classification). For those small sample data we used the fisher's exact test. Using independent-samples T test to compare the age. The Kaplan-Meier method was used to calculate the OS,LFFS and DMFS rates. The log-rank test was used to compare the statistical differences in endpoints in both groups. Using Cox proportional hazards model to calculate the Multivariate analysis. All statistical tests were two-sided, and P<0.05 was considered statistically significant .

## RESULTS

### Patient outcomes

#### OS

**Figure 1:**
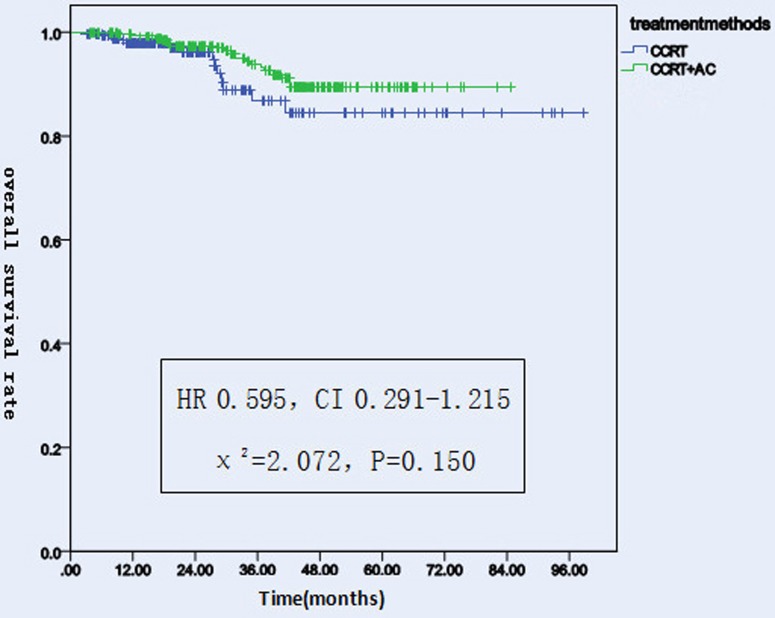
Overall survival rates of 522 NPC patients treated with different methods

Before matching, there were 58 patients dead in the whole group, including 14 of the CCRT group and 44 of the CCRT+AC group. After matching, there were 32 patients dead in the whole group, including 14 patients in the CCRT group and 18 in the CCRT+AC group. The 1-,2-,3-year overall survival rates were 97.9%, 96.2%, 86.9%(95% CI 82-94),respectively in CCRT group versus 99.6%, 97.4%, 93.8%(95% CI 77-82) in CCRT+AC group. There were no significant difference in OS between the two groups (HR 0.595, CI 0.291-1.215, χ^2^ = 2.072, *P* = 0.150) (Figure 1 and 4). After stratification by disease stage[UICC(7th edition)], the 1,2 and 3 years OS rates were all 100.0% in both groups for stage II. For stage III, the the 1,2 and 3 years os rates were 100.0%,95.8%,84.0% in CCRT group, while in CCRT+AC group,the 1-,2- and 3- year os rates were 100.0%, 97.6%,94.4%. For stage IV, the 1-,2- and 3- year os were 95.4%,95.4%,79.4%,respectively in CCRT group versus 99.1%, 96.3%,90.5% in CCRT+AC group. There were statistically significant difference in the whole group(HR 0.482, CI 0.232-1.004, χ^2^ = 3.931,*P* = 0.047).However, after we pairwise for each stratum, there were no statistically significant difference for stage III and IV in 1-,2- and 3- year os rates (χ^2^ = 1.811, *P* = 0.178; χ^2^ = 2.168, *P* = 0.141)between the two groups. For T1,the 1-,2- and 3- year os rates were all 100.0% between the two groups. For T2, the 1-,2- and 3- year os rates were 100.0%, 96.7%, 96.7% in CCRT group versus 100.0%, 98.0%, 95.5% in CCRT+AC group. For T3,the 1,2,and 3 years os rates were 100.0%, 97.1%, 80.9% in CCRT group, while in CCRT+AC group the 1,2,and 3 years os rates were 100.0%, 96.9%, 95.0%.For T4,the 1,2,and 3 years os rates were 95.2%, 95.2%, 78.2% in CCRT group versus 99.1%, 97.1%, 90.9% in CCRT+AC group. There were no significant difference in os of the whole group (HR 0.494, CI 0.238-1.027, χ^2^ = 3.682, *P* = 0.055).For N0, there were 100.0% of the 1,2,3 years os rates in the two groups. For N1,the 1-, 2-, 3- year os rates were 96.5%, 94.3%, 94.3% in CCRT group, while in CCRT+AC group there were 100.0%,100.0%,98.7%,respectively. For N2, the 1-, 2-, 3- year os rates were 98.4%, 96.8%, 75.4% in CCRT group versus 99.2%, 95.9%, 88.9% in the CCRT+AC group. For N3,the 1-, 2-, 3- year os rates were 100.0% in CCRT group versus 100.0%, 90.9%, 90.9% in CCRT+AC group. There were no significant difference in the whole group (HR 0.543, CI 0.263-1.121, χ^2^ = 2.803, *P* = 0.094, Table 5).

#### LFFS

**Figure 2:**
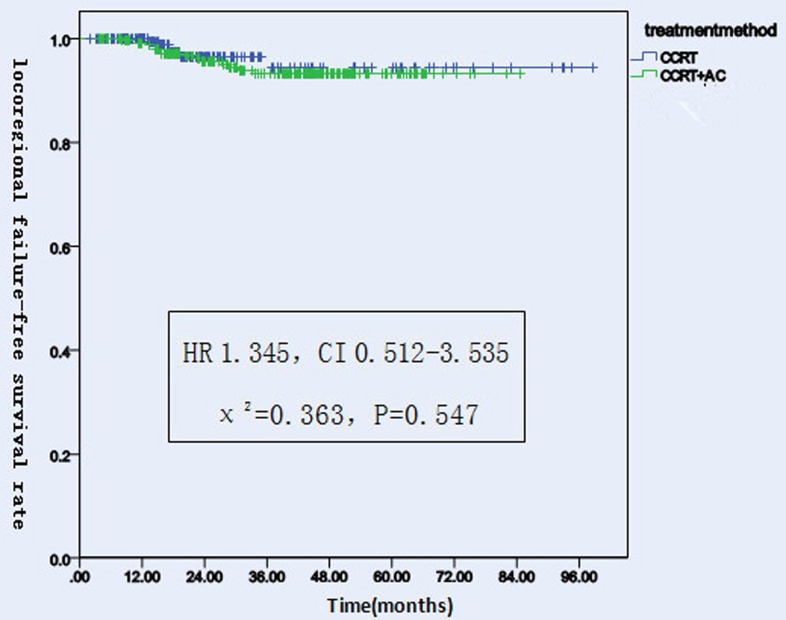
Locoregional failure-free survival rates of 522 NPC patients treated with different methods .

There were 20 patients had localregional failure, including 6 patients in CCRT group and 14 patients in CCRT+AC group. Among them, 5 patients relapsed at the neck, the others all relapsed at nasopharynx . The 1, 2, 3 years LFFS rates were 100.0%, 96.5%, 96.5%(95% CI 91-98) in the CCRT group versus 99.2%, 95.6%, 93.2% (95% CI 78-83)in the CCRT+AC group. There were no significant difference between the two groups(HR 1.345, CI 0.512-3.535, χ^2^ = 0.363, *P* = 0.547) (Figure 2 and 5). After stratification by disease stage[UICC(7th edition)]. In CCRT group,the 1,2 and 3 years LFFS rates were all 100.0% for stage II, 100.0%, 96.2%, 96.2%, for stage III, 100.0%, 95.3%, 95.3% for stage IV,respectively .On the contrary, in CCRT+AC group, the 1-,2-,3-year LFFS rates were all 100.0% for stage II, 100.0%, 97.7%, 96.1% for stage III, 98.2%, 92.1%, 87.7% for stage IV,respectively. There were no statistically significant difference in the whole group (HR 1.187, CI 0.446-3.160, χ^2^ = 0.118, *P* = 0.731). For T1, both groups were 100.0% of the 1-, 2-, and 3-year LFFS rates. For T2,the 1-, 2-, and 3-year LFFS rates were all 100.0% in CCRT group versus 100.0%, 98.1%, 98.1% in CCRT+AC group. For T3, the 1-, 2-, and 3-year LFFS rates were 100.0%, 95.1%, 95.1% in CCRT group versus 98.6%, 97.0%, 92.7% in CCRT+AC group. For T4, the 1,2 and 3 years LFFS rates were 100.0%, 95.1%,95.1% in CCRT group,respectively. While in CCRT+AC group there were 99.1%, 92.5%, 89.6%, respectively. There were no statistically significant difference in the whole group (HR 1.207, CI 0.455-3.204, χ^2^ = 0.143, *P* = 0.705). In CCRT group ,there were 100.0%, 100.0%,100.0% of the 1-,2-,3-year LFFS rates for N0. 100.0%, 98.4%, 98.4%for N1. 100.0%, 94.5%, 94.5% for N2. 100.0%, 100.0%,100.0% for N3. In CCRT+AC group, the 1-,2-,3-year LFFS rates were all 100.0% for N0. 100.0%, 97.6%, 96.3% for N1. 99.3%, 94.3%, 91.7% for N2. 91.7%, 91.7%, 68.8% for N3.There were no significant difference in the whole group(HR 1.301, CI 0.489-3.462, χ^2^ = 0.279, *P* = 0.597, Table 5).

**Table 4:** multivariate Cox regression analysis of the prognostic factors

	OS				LFFS				DMFS			
	HR	95%CI		P value	HR	95%CI		P value	HR	95%CI		P value
		Lower	upper			Lower	upper			Lower	upper	
**Age**	1.013	0.981	1.040	0.423	0.977	0.939	1.018	0.271	0.994	0.967	1.023	0.702
**KPS**	0.777	0.356	1.697	0.526	0.753	0.268	2.117	0.590	0.770	0.380	1.559	0.468
**Overall Stage**	2.007	0.550	7.325	0.291	1.896	0.366	9.820	0.446	1.684	0.599	4.732	0.323
**Course Of con-chemo^a^**	0.588	0.329	1.050	0.073	0.882	0.421	1.849	0.740	0.527	0.327	0.848	0.008
**Course Of adj-chemo^b^**	0.814	0.567	1.166	0.261	1.096	0.697	1.722	0.692	0.968	0.719	1.304	0.830

a=Course Of con-chemotherapy

b= Course of adjuvant chemotherapy

**Table 5:** The results from Stratified analysis in CCRT group and CCRT+AC group a By Union for International Cancer Control criteria (7th edition).

Stage ^a^	OS(%)						
	1 year		2 year		3 year		
	CCRT	CCRT+AC	CCRT	CCRT+AC	CCRT	CCRT+AC	P value
Overall							0.150
	97.9	99.6	96.2	97.4	86.9	93.8	
Classification ^a^						0.047	
II	100.0	100.0	100.0	100.0	100.0	100.0	
III	100.0	100.0	95.8	97.6	84.0	94.4	
IV	95.4	99.1	95.4	96.3	79.4	90.5	
T classification^a^							0.055
T1	100.0	100.0	100.0	100.0	100.0	100.0	
T2	100.0	100.0	96.7	98.0	96.7	95.5	
T3	100.0	100.0	97.1	96.9	80.9	95.0	
T4	95.2	99.1	95.2	97.1	78.2	90.9	
N classification^a^							0.094
N0	100.0	100.0	100.0	100.0	100.0	100.0	
N1	96.5	100.0	94.3	100.0	94.3	98.7	
N2	98.4	99.2	96.8	95.9	75.4	88.9	
N3	100.0	100.0	100.0	90.9	100.0	90.9	
**Stage ^a^**	**LFFS(%)**						
	**1 year**		**2 year**		**3 year**		
	**CCRT**	**CCRT+AC**	**CCRT**	**CCRT+AC**	**CCRT**	**CCRT+AC**	**P value**
Overall							0.547
	100.0	99.2	96.5	95.6	96.5	93.2	
Classification ^a^							0.731
II	100.0	100.0	100.0	100.0	100.0	100.0	
III	100.0	100.0	96.2	97.7	96.2	96.1	
IV	100.0	98.2	95.3	92.1	95.3	87.7	
T classification^a^							0.705
T1	100.0	100.0	100.0	100.0	100.0	100.0	
T2	100.0	100.0	100.0	98.1	100.0	98.1	
T3	100.0	98.6	95.1	97.0	95.1	92.7	
T4	100.0	99.1	95.1	92.5	95.1	89.6	
N classification^a^							0.597
N0	100.0	100.0	100.0	100.0	100.0	100.0	
N1	100.0	100.0	98.4	97.6	98.4	96.3	
N2	100.0	99.3	94.5	94.3	94.5	91.7	
N3	100.0	91.7	100.0	91.7	100.0	68.8	
**Stage ^a^**	**DMFS(%)**						
	**1 year**		**2 year**		**3 year**		
	**CCRT**	**CCRT+AC**	**CCRT**	**CCRT+AC**	**CCRT**	**CCRT+AC**	**P value**
Overall							0.350
	97.4	95.3	92.8	91.8	89.0	87.2	
Classification ^a^							0.439
II	100.0	100.0	100.0	100.0	94.7	100.0	
III	95.8	98.0	89.6	95.8	89.6	92.6	
IV	97.9	91.5	92.7	85.6	84.9	77.1	
T classification^a^							0.420
T1	100.0	100.0	100.0	100.0	100.0	90.9	
T2	97.9	98.2	97.9	96.3	91.3	94.1	
T3	95.9	95.9	87.7	94.5	87.7	92.8	
T4	97.8	92.8	92.3	86.5	83.9	78.8	
N classification^a^							0.347
N0	100.0	100.0	100.0	100.0	100.0	100.0	
N1	96.6	97.9	96.6	97.9	92.8	95.3	
N2	97.5	94.0	88.6	87.4	84.3	82.0	
N3	100.0	84.6	100.0	84.6	100.0	50.8	

a = By Union for International Cancer Control criteria (7th edition) Abbreviations: OS, overall survival; LFFS, locoregional failure-free survival; DMFS, distant metastasis failure-free survival; CCRT, concurrent chemoradiotherapy; AC, adjuvant chemotherapy

#### DMFS

**Figure 3:**
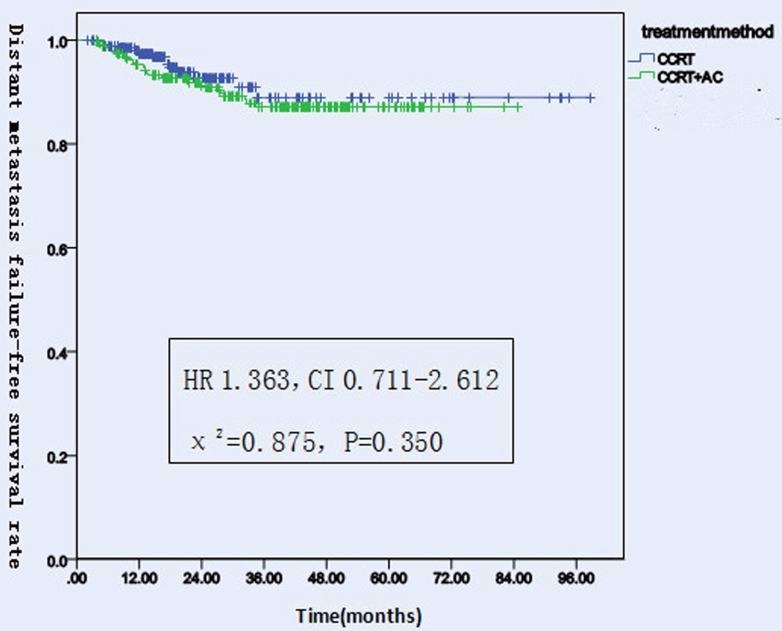
Distant metastasis failure-free survival rates of 522 NPC patients treated with different methods .

There were 42 patients had distant metastasis, including 14 patients in CCRT group and 28 patients in CCRT+AC group. Among them, there were 12 cases had distant metastasis in lung,9 cases in bone, 6 cases in brain, bone and lung,5 cases both in lung and bone,5 cases in liver,1 case both in lung and liver,1 case in bone and brain,2 cases in lung, bone and liver (Table 6) . The 1-,2-,3-year MDFS rates were 97.4%, 92.8%, 89.0% (95% CI 85-95)in CCRT group. While in CCRT+AC group the 1-,2-,3-year DMFS rates were 95.3%, 91.8%, 87.2%(95% CI 73-79).There were no significantly difference in the two groups (HR 1.363, CI 0.711-2.612, χ^2^ = 0.875, *P* = 0.350, Figure 3 and 6). After stratification by disease stage[UICC(7th edition)], the 1,2,and 3 years DMFS rates were 100.0%, 100.0%, 94.7% in CCRT group versus 100.0%, 100.0%, 100.0% in CCRT+AC group For stage II. For stage III,the 1,2,and 3 years DMFS rates were 95.8%, 89.6%, 89.6% in CCRT group versus 98.0%, 95.8%, 92.6% in CCRT+AC group. For stage IV,the 1,2,and 3 years DMFS rates were 97.9%, 92.7%, 84.9% in CCRT group versus 91.5%, 85.6%, 77.1% in CCRT+AC group. There were no statistically significant difference in the whole group(HR 1.295, CI 0.672-2.495, χ^2^ = 0.600, *P* = 0.439).In CCRT group, the1,2,and 3 years DMFS rates were all 100.0% for T1.97.9%, 97.9%, 91.3% for T2. 95.9%, 87.7%, 87.7% for T3. 97.8%, 92.3%, 83.9% for T4,respectively.While in the CCRT+AC group, the comparable rates were 100.0%, 100.0%, 90.9% for T1. 98.2%, 96.3%, 94.1% for T2. 95.9%, 94.5%, 92.8% for T3. 92.8%, 86.5%, 78.8% for T4,respectively. There were no statistically significant difference in the results (HR 1.309, CI 0.680-2.521, χ^2^ = 0.651, *P* = 0.420). In CCRT group, the 1,2,and 3 years DMFS rates were all 100.0% for N0. 96.6%, 96.6%, 92.8% for N1. 97.5%, 88.6%, 84.3% for N2.100.0%,100.0%,100.0% for N3.But in CCRT+AC group, the comparable rates were all 100.0% for N0. 97.9%, 97.9%, 95.3% for N1. 94.0%, 87.4%, 82.0% for N2. 84.6%, 84.6%, 50.8% for N3,respectively.There were no significantly difference in that results (HR 1.366, CI 0.711-2.623, χ^2^ = 0.883, *P* = 0.347, Table 5).

**Figure 4:**
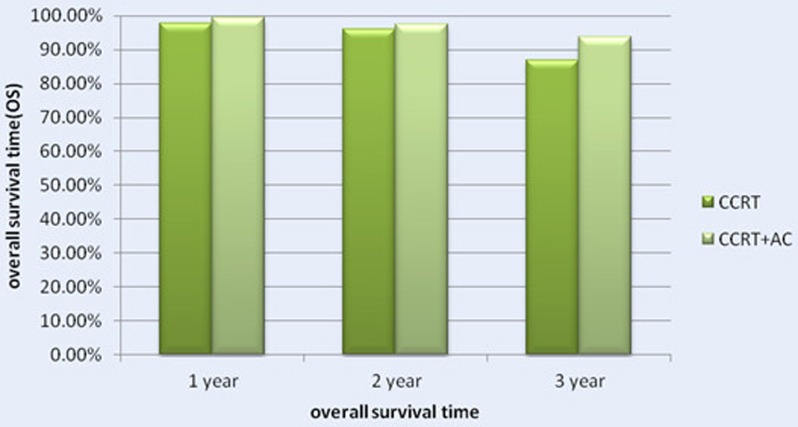
The 1-, 2- and 3- Year Overall Survival Rate (OS) for Patients with CCRT in Comparison with CCRT + AC for NPC patients .

**Figure 5:**
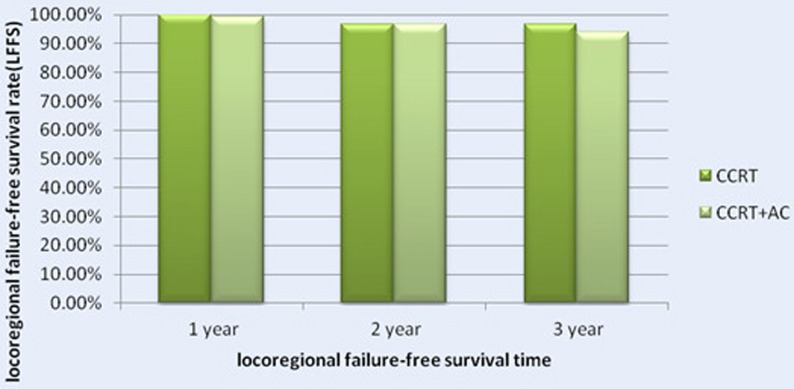
The 1-, 2- and 3- Year locoregional failure-free survival rate (LFFS) for Patients with CCRT in Comparison with CCRT+AC for NPC patients .

**Figure 6:**
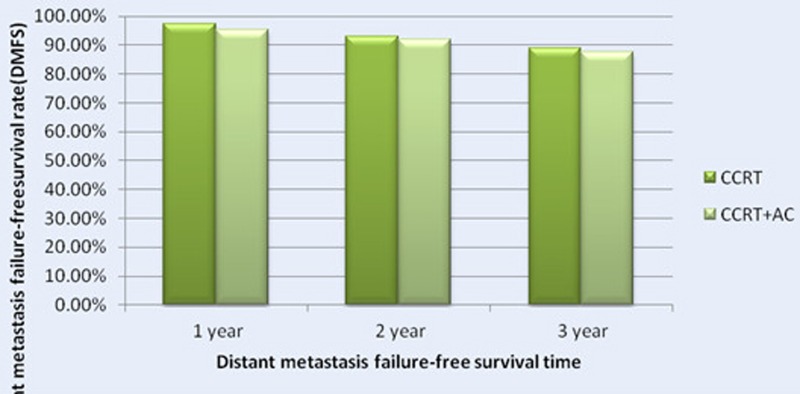
The 1-, 2- and 3- Year distant metastasis failure-free survival rate (DMFS) for Patients with CCRT in Comparison with CCRT+AC for NPC patients .

**Table 6:** The distribution of 42 NPC patients with distant metastasis

distant metastasis	CCRT (n=14)	CCRT+AC (n=28)
lung	5	7
bone	2	7
brain, bone and lung	2	4
lung and bone	1	4
liver	2	3
lung and liver	1	0
bone and liver	0	1
bone and brain	1	0
lung, bone and liver	0	2

### Multivariate analyses

Multivariate Analyses by Cox proportional hazards model revealed that age, KPS scores, overall stage, treatment method and chemotherapy regimen were not the prognostic factors for OS. Only course of the concurrent chemotherapy was the prognostic factors for DMSF(Table 4).

## DISCUSSION

In the age of two-dimensional radiotherapy, the five-year overall survival rate had a good efficiency in NPC [5], but it brought heavier sequelae and more chances to get radio-encephalopathy, patients had low quality of life. Since intensity modulated radiotherapy technique arose, because its targeted volume is closer to the tumor's shape, and it has lower injury to proximal critical normal tissues. So it gradually replace the two-dimensional radiotherapy, becoming a principal treatment method for NPC patients. Zhang et al [6] made a study in the application of two-dimensional radiotherapy and IMRT in NPC patients, the results showed that using IMRT can obviously relieved acute or chronic radiation damage, and it had lower chance to have radio-sequelae than using two-dimensional radiotherapy, but IMRT could not improve distant metastasis-free rate, failure-free survival rate and overall survival rate. Later, Lai et al. [7] make a comparison between IMRT and two-dimensional radiotherapy in NPC. They found that IMRT significantly improved the treatment efficacy, patients achieved a good regional control rate, especially for those patients who had early T stage. In 2004, a multi-institutional survey of the effectiveness of chemotherapy plus radiotherapy for NPC by Mitsuhiko Kawashima [8] showed that, concurrent chemotherapy combine with radiotherapy could significantly improved the overall survival rate than only received radiotherapy in advanced NPC patients. A great deal of studies [9, 10, 20] had approved concurrent chemoradiotherapy had advantages in improving overall survival rate and progression free survival rate, what indicated that locally advanced NPC patients received concurrent chemoradiotherapy would more beneficial. So in 2010, National Comprehensive Cancer Network (NCCN) recommended concurrent chemoradiotherapy was the standard treatment method for NPC. In 2014, Tingting Xu et al [11] make a comparison between concurrent chemoradiotherapy and neoadjuvant chemotherapy for locally advanced NPC patients. The results pointed out both regimens showed similar efficacy. Only concurrent chemotherapy could ameliorate distant metastasis free survival rate for those stage T3-4N0-1 NPC patients. In a trail by su [12], 865 NPC patients received IMRT alone or plus chemotherapy, they classified the candidates into four groups, From the study they made a conclusion that the early disease group had the lowest treatment failure rate. For those NPC patients with early stage, IMRT alone could produce satisfactory results. While for those patients who had locally advanced, chemoradiotherapy was better. Early in 1988, Rossi A et al. [13] had a comparison between radiotherapy plus adjuvant chemotherapy and radiotherapy alone. They found there were no significantly difference in two-year progression-free survival rate and the four-year overall survival rate (55.8% vs. 57.7%, 67.3% vs. 58.5%). In 2010, Kyong Hwa Park et al. [14] reported that the overall response rate of CCRT was 95%, after used AC, the overall response rate was 100%. So they make a conclusion that using cisplatin and 5-FU in combination with radiotherapy followed by three cycles of BEC chemotherapy was effective in locally advanced NPC patients. In 2011,a study by Anne W.M. Lee et al. [15] showed that, concurrent chemoradiotherapy followed by adjuvant chemotherapy can significantly reduced treatment failure and cancer-specific deaths. Recently, a meta analysis by Marie Yan showed that concurrent chemoradiotherapy followed by adjuvant chemotherapy can't enhance survival rates. An other meta-analysis at the same year by Blanchard P et al. [4] found that, concurrent chemoradiotherapy were better than chemotherapy alone in improving survival rates. In our study, no significantly difference in 1-,2-,3-year OS, LFFS and DMFS between CCRT alone and CCRT+AC in NPC patients(χ^2^ = 2.072, *P* = 0.150; χ^2^ = 0.363, *P* = 0.547; χ^2^ = 0.875, *P* = 0.350),what are similar to the results by Dora L.W. et al. [16] in 2004.Nevertheless, after stratification by disease stage, we found a statistically significant difference in OS(*P* = 0.047). But after we pairwise for each stratum, there were no statistical significance for stage III and IV in two-year os rates (χ^2^ = 1.811, *P* = 0.178; χ^2^ = 2.168, *P* = 0.141). We thought it was due to confounding factors. Although other results from the Stratified analysis didn't make a statistically significant difference, we saw a trend that the CCRT+AC group has higher OS than CCRT group for locally advanced NPC(III,IV and T4)patients from the results .Adding adjuvant chemotherapy for locally advanced NPC patients may benefit. Furthermore,addition of AC couldn't improved the 1-,2-,3-year LFFS and DMFS for N2-3. On the contrary, it may reduced the 1-,2-,3-year LFFS rate or DMFS rate .This results compared with a study reported by liang [19] at 2014 has some inconsistencies .He reported that concurrent chemoradiotherapy plus adjuvant chemotherapy didn't significantly improve the 2-year OS,LFFS,DMFS, but he found a borderline significant difference in os by CCRT+AC treatment in patients with N2-3 disease(*P* = 0.052).Analyzed its cause, the main possibility is we enlarged the number of participants. Why concurrent chemoradiotherapy followed by adjuvant chemotherapy may reduced the efficacy for N2-3 patients? One possibility is that for those advanced nodal NPC patients(N2-3), concurrent chemoradiotherapy also have a better efficacy, after chemoradiotherapy, local organizations fibrosis so that with poor blood supply, chemotherapy drugs hardly get into the tumor site, so the plasma concentration beyond the reach of effective concentration. Another possibility explanation is that stage N2-3 patients have poor immunity so they can't tolerant the chemotherapy. In a phase 3 multicentre randomised controlled trial reported by chen et al. [17], concurrent chemoradiotherapy followed by cisplatin and fluorouracil chemotherapy did not improve failure-free survival rate in locoregionally advanced NPC patients. The same conclusion by Shiping Yang [18], concurrent chemoradiotherapy plus adjuvant cisplatin or nedaplatin and 5-fluorouracil chemotherapy didn't significantly improve 3 years OS, LRFS,FFS,DMFS. In 2011, a trail reported by Anne W.M. Lee [21], concurrent chemoradiotherapy was important to locoregional control and survival. Moreover, they though 2 concurrent cycles of cisplatin were enough. Adjuvant chemotherapy using fluorouracil-containing combination can improved distant control. At present, it is uncertain whether concurrent chemoradiotherapy followed by chemotherapy have survival benefit.

Although our trail is a big data retrospective analysis, it has several limitations. The reliability needs more prospective studies to verify the conclusion.

## CONCLUSIONS

From this study, IMRT combined with concurrent chemotherapy followed by adjuvant chemotherapy in NPC might improve the 1-,2-,and 3- year OS, but no significant difference were observed between the two groups. There was no statistically significant difference in LFFS and DMFS. For those locally advanced NPC patients(III,IV and T4) might benefit from adjuvant chemotherapy .
